# Early‐life predictors and risk factors of peanut allergy, and its association with asthma in later‐life: Population‐based birth cohort study

**DOI:** 10.1111/cea.14103

**Published:** 2022-02-10

**Authors:** Constantinos Kotsapas, Nicolaos Nicolaou, Sadia Haider, Gina Kerry, Paul J. Turner, Clare S. Murray, Angela Simpson, Adnan Custovic

**Affiliations:** ^1^ 4615 National Heart and Lung Institute Imperial College London London UK; ^2^ 486462 University of Nicosia Medical School Nicosia Cyprus; ^3^ Division of Infection, Immunity and Respiratory Medicine School of Biological Sciences Faculty of Biology, Medicine and Health University of Manchester, and Manchester Academic Health Science Centre and NIHR Biomedical Research Centre Manchester University Hospitals NHS Foundation Trust Manchester UK

**Keywords:** asthma, atopic dermatitis, epidemiology, *filaggrin*, food allergy, genetics, peanut

## Abstract

**Background:**

Understanding risk factors for peanut allergy (PA) is essential to develop effective preventive measures.

**Objective:**

The objective was to ascertain associates and predictors of PA, and the relationship between PA and asthma severity.

**Methods:**

In a population‐based birth cohort, we investigated the association between objectively confirmed PA with early‐life environmental exposures, *filaggrin* (*FLG*)‐loss‐of‐function mutations and other atopic disease. We then examined the association of PA with longitudinal trajectories of sensitization, wheeze and allergic comorbidities, which were previously derived using machine learning. Finally, we ascertained the relationship between PA and asthma severity.

**Results:**

PA was confirmed in 30/959 participants with evaluable data. In the multivariate analysis, eczema in infancy (OR = 4.4, 95% CI 1.5–13.2, *p* = 0.007), egg sensitization at age 3 years (OR = 9.7, 95% CI 3.3–29.9, *p *< 0.001) and early‐life cat ownership (OR = 3.0, 95% CI 1.1–8.4, *p* = 0.04) were independent associates of PA. In the stratified analysis among 700 participants with genetic information, in children with early‐life eczema there was no difference in *FLG* mutations between children with and without PA (3/18 [16.7%] vs. 42/220 [19.1%], *p* = 1.00). In contrast, among children without eczema, those with PA were almost eight times more likely to have *FLG* mutations (2/6 [33.3%] vs. 27/456 [5.9%], *p* = 0.049). We observed associations between PA and multiple allergic sensitization profiles derived using machine learning, with ~60‐fold increase in risk among individuals assigned to multiple early sensitization. PA was significantly associated with persistent wheeze (but not other wheeze phenotypes), and with trajectories of atopic disease characterized by co‐morbid persistent eczema and wheeze (but not with transient phenotypes). Children with PA were more likely to have asthma, but among asthmatics we found no evidence of an association between PA and asthma severity.

**Conclusions:**

Peanut allergy is associated with multiple IgE sensitization and early‐onset persistent eczema and wheeze. *FLG* loss‐of‐function mutations were associated with peanut allergy in children without eczema.

## Graphical abstract

Peanut allergy is associated with multiple IgE‐sensitisation and early‐onset persistent eczema and wheeze. FLG loss‐of‐function mutations were associated with peanut allergy in children without eczema.

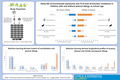

**Key Messages**

- Peanut Allergy is associated with longitudinal trajectories characterized by early‐onset and persistent eczema and wheeze.
- Among children without eczema, *FLG* mutations are significantly associated with peanut allergy.
- Peanut‐allergic children are more likely to have asthma, but among asthmatics, peanut allergy is not associated with more severe asthma.

## 1 INTRODUCTION

Peanut allergy is among the most common food allergies, affecting ~2% of children and 0.5% of adults in the UK,^1^, ^2^, ^3^ and ~2% of <18‐year‐olds in the United States.^4^ Peanut allergy tends to start in early life and has generally been considered to be lifelong,^5^ although some studies suggest that >20% of children may outgrow it.^6^ Data from the United States suggest that prevalence of peanut allergy has increased in the first decade of 21st century,^7^ and studies looking at nationwide UK records between 2001 and 2005 revealed similar trends.^8^ Reactions in peanut‐allergic individuals can be unpredictable and severe, and although death remains rare, peanut allergy is among the most common causes for food‐related fatal anaphylaxis.^9^, ^10^ In patients with food allergy, a history of asthma and previous asthma exacerbations are risk factors for fatal anaphylaxis.^11^, ^12^ Among asthmatic patients, the presence of peanut allergy is often considered to be associated with more severe asthma,^13^ but the data about this relationship are sparse.^14^

The mainstay of management is strict avoidance and carrying of adrenaline auto‐injectors for emergency use.^15^ Avoidance, however, can be difficult,^16^ and accidental reactions are relatively common.^17^ This creates anxiety among patients and their families, leading to restrictions in eating habits and social activities.^18^ Peanut oral immunotherapy offers promise for treatment, but this approach focusses on mitigating the risk of reactions due to accidental exposure rather than providing a cure.^19^, ^20^ Measures aimed at primary and secondary prevention remain the preferred option for addressing this growing problem,^21^ highlighting the importance of understanding the risk factors and predictors of peanut allergy.

Various risk factors for food and peanut allergy were suggested in previous studies (summarized in Table S1). These include non‐modifiable factors such as male sex^8^ and ethnicity (Afro‐Caribbean or Indian background),^22^, ^23^ but also potentially modifiable factors such as altered microbial exposures,^24^ diet,^25^ the presence of other allergic diseases, in particular eczema,^26^ and the timing, route and dose of allergen exposures.^27^ Genetic associates of peanut allergy such as the *filaggrin* (*FLG*) loss‐of‐function mutations have also been reported.^28^ However, although population‐based birth cohort is the optimal study design to investigate risk factors for a disease such as peanut allergy, there is a paucity of data on the associates of oral food challenge (OFC)‐confirmed peanut allergy in unselected birth cohorts (Table S1).

We capitalized on the availability of objective data on peanut allergy in the Manchester Asthma and Allergy Study (MAAS), a population‐based birth cohort, in which OFCs to peanut were carried out in school‐aged peanut‐sensitized participants at the age 9–10 years.^2^, ^29^, ^30^ This study enabled identification of specific IgE (sIgE) to the peanut component Ara h 2 as an important biomarker of peanut allergy^2^, ^29^ and provided a setting to show that increasing early‐life domestic exposure to peanut allergen in household dust increases the likelihood of subsequent peanut allergy in a dose–response fashion among children with *FLG* mutations, but not in those with *FLG* wild‐type genotype.^30^ This gene–environment interaction remained significant after adjusting for infantile eczema, suggesting that the effect of *FLG* genotype may be independent of eczema.^30^ Furthermore, application of machine‐learning and data‐driven methods to longitudinally collected data in this cohort enabled discoveries of different eczema and wheeze phenotypes,^31^, ^32^ different subtypes of allergic sensitization,^33^ and developmental trajectories and co‐occurrence of eczema, wheeze and rhinitis.^34^ Our recent study highlighted potential importance of such data‐driven phenotypes in relation to peanut allergy by showing that different longitudinal trajectories of eczema differ in their association with comorbidities, including a 9‐ to 11‐fold increase in the risk of peanut allergy among children in the persistent eczema, but not other eczema classes.^31^

In the current analysis, we aimed to ascertain early‐life predictors of peanut allergy and address a specific hypothesis that early‐life eczema and *FLG* loss‐of‐function mutations are independent risk factors. We then proceeded to investigate the association between previously derived longitudinal trajectories of sensitization,^33^ wheeze^32^ and allergic comorbidities,^34^ and peanut allergy. Finally, we focussed on the relationship between peanut allergy and asthma severity, to investigate the notion that co‐morbid peanut allergy in asthmatic patients is associated with more severe asthma.^13^

## 2 METHODS

### 2.1 Study design, setting and participants

Manchester Asthma and Allergy Study is a population‐based birth cohort.^35^, ^36^ Detailed description is provided in the Supplementary Appendix. Briefly, participants were recruited prenatally and followed prospectively, attending review clinics at ages 1, 3, 5, 8 and 11 years. The study received approval by the local ethics committee, and informed consent was obtained in all cases.

### 2.2 Data sources

#### 2.2.1 Questionnaires

At each visit, interviewer‐administered validated questionnaires^37^, ^38^ were used to collect information on environmental exposures, parentally reported symptoms, physician‐diagnosed diseases and treatments received. These were enriched with additional questions on food allergy and diet.^39^

#### 2.2.2 Allergic sensitization

We completed skin prick tests (SPTs) and sIgE measurement (ImmunoCAP^™^, Phadia) to common food (milk, egg and peanut) and aero‐allergens (house dust mite‐HDM, cat, dog, moulds, grass and birch pollen) at all time‐points.

Component resolved diagnostics (CRD) to peanut components Ara h 1–3 was performed by ImmunoCAP (age 8 years).^29^ Further CRD data were obtained through multiplex Immuno Solid‐Phase Allergen Chip (ISAC).^40^

#### 2.2.3 Primary care medical records

We transcribed data from primary healthcare records for the first 10 years of life^41^ (details in Supplementary Appendix). This included information on asthma medication and oral corticosteroid prescriptions, wheeze episode presentations to the GP, and asthma/wheeze related emergency department attendances and hospital admissions.

#### 2.2.4 Genotyping

*Filaggrin (FLG)* genotyping was performed as previously described^30^, ^42^ to ascertain the presence of six *FLG* loss‐of‐function mutations (R501X, S3247X, R2447X, 2282del4, 3673delC and 3702delG).

### 2.3 Definitions of outcomes and key variables

#### 2.3.1 Peanut sensitization

Skin prick test mean wheal diameter (MWD) to peanut extract of 3 mm or greater than the negative control and/or sIgE to whole peanut extract >0.35 kU_A_/L.

#### 2.3.2 Peanut allergy

All cohort children with evidence of peanut sensitization at ages 5 and/or 8 years (SPT response ≥3 mm or sIgE level ≥0.2 kU_A_/L), or history of immediate reaction upon exposure, were offered an OFC to confirm peanut allergy.^2^, ^29^, ^30^ To enrich peanut‐allergic group, we recruited 12 children (6 boys) with confirmed peanut allergy from a tertiary referral local allergy clinic (reaction on exposure to peanut and sIgE ≥ 15 kUa/L and/or SPT ≥ 8 mm)^2^; these children had longitudinally collected data to facilitate studies on peanut allergy and did not undergo OFC. Detailed description of OFC protocol is provided in the Supplementary Appendix. Briefly, open OFCs were carried out among children who had a history of tolerating peanut on consumption; all other children underwent a double‐blind, placebo‐controlled food challenge (DBPCFC). Two or more objective signs were required to constitute a positive challenge.^2^, ^30^

Non‐sensitized children with no history of a previous reaction who were consuming peanuts were *a priori* classified as non‐peanut allergic. Children with negative peanut OFC were also classified as peanut non‐allergic.

#### 2.3.3 FLG loss‐of‐function mutations

Children carrying any one or more of the six mutations were defined as carriers.^30^, ^42^

Definitions of all other variables (including previously derived data‐driven longitudinal patterns of allergic sensitization,^33^, ^43^ wheeze phenotypes^32^ and atopic multimorbidity clusters^34^, ^44^) are presented in the Supplemental appendix.

### 2.4 Statistical analysis

All evaluable data were used in the analysis. Differences between groups were assessed using unpaired t‐test for normally distributed continuous data, Mann–Whitney for skewed continuous data and Pearson's chi‐square and Fisher's exact test for categorical binary data. Univariate logistic regression analysis was carried out to assess the unadjusted odds ratio (OR) of each independent variable to the outcome. Multivariate logistic regression models were then used to examine the association of each independent variable with peanut allergy; results are reported as ORs and 95% confidence intervals (CI). Variables for the multivariate analysis were selected based on the findings in the univariate analyses and the results of previous studies. We used STATA 14.2 for all analyses.

## 3 RESULTS

We included 959 children in this analysis (514 [53.6%] boys and 895 [93.3%] White‐Caucasian). Demographic characteristics of the study population and comparisons between included and excluded participants are presented in Table S2. In the included group, we observed higher breastfeeding rates (71.7% vs. 61.9%; *p *= 0.007) and day‐care attendance (70.0% vs. 59.6%; *p *= 0.005), and lower proportion of older siblings (53.6% vs. 71.5%; *p *< 0.001), maternal smoking (13.5% vs. 20.2%, *p *= 0.012) and non‐working parents (2.6% vs. 8.9%; *p *= 0.004).

### 3.1 Peanut allergy and tolerance in the study population

**FIGURE 1:**
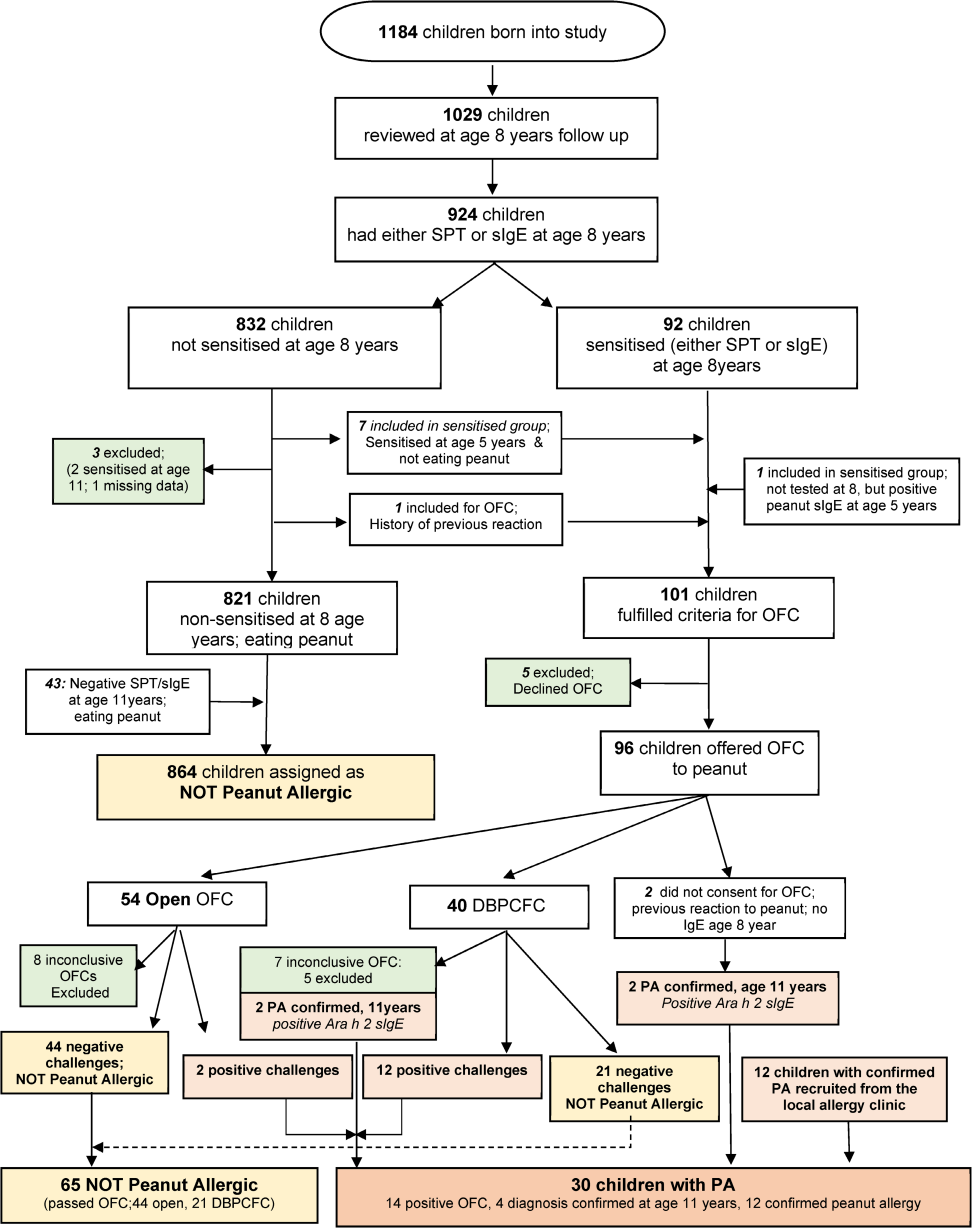
Participant flow

Figure 1 shows the participant flow. Of 1029 children who attended follow‐up at age 8 years, 864 were classified as not allergic to peanut (no peanut sensitization at ages 5 and/or 8, eating peanuts, no reactions); 924/1029 had either SPT or sIgE at age 8 years, of whom 101 fulfilled criteria for further study to confirm/refute peanut allergy (100 peanut‐sensitized, one not sensitized, but reported reaction upon exposure) and were offered OFC, of whom 5 declined. Of the 96 participants who agreed to take part, 2 withdrew the consent for OFC and were subsequently classified as peanut allergic based on additional data on positive sIgE to Ara h 2 (>0.35 kuA/L measured by ImmunoCAP and >0.3 ISU measured by ISAC chip at age 11 years). Of 94 children who underwent OFC (40 DBPCFC, 54 open), 14 had positive OFC, 65 passed OFC, and 15 had inconclusive OFC (of those, 2 were classified as peanut allergic based on IgE sensitization to Ara h 2, and 13 were excluded). Detailed information on 4 children who were diagnosed as peanut allergic based on sIgE to Ara h 2 is presented in Supplementary Appendix. Children (*n* = 12) recruited from local allergy clinic with severe reaction upon exposure, SPT ≥ 8 mm and/or IgE ≥ 15 kuA/L) were *a priori* classified as peanut allergic. Thereby, in total, 30 children in this analysis had confirmed peanut allergy, and 929 were not allergic to peanut (864 not sensitized and eating peanuts, and 65 who were sensitized, but had negative OFC).

### 3.2 Characteristics of peanut‐allergic children and those not allergic to peanut

Table 1 shows the demographic and early‐life characteristics of peanut‐allergic and non‐allergic children. Peanut allergy was significantly associated with parentally reported eczema in the first year of life, any sensitization (SPT) at 1 and 3 years and egg sensitization at 1 and at 3 years. We observed marginal differences with male sex, parental atopy, parental asthma and cat ownership during pregnancy, but these differences did not reach statistical significance. Similarly, small differences between the groups were noted in the rate of reported wheeze in infancy and rhinitis at age 3 years. Peanut‐allergic children were almost 2 times more likely to have *FLG* mutations (20% vs. 10.4%), but this difference was not statically significant due to issues related to sample size.

**TABLE 1:** Comparison of demographic and early‐life characteristics between peanut‐allergic (PA) and peanut non‐allergic (Not PA) children

Variables: Categorical (proportions, %) Numerical (mean, SD)	Study population (*n* = 959)	No peanut allergy (*n* = 929)	Peanut allergy (*n* = 30)	*p*‐Value	Odds ratio (95% CI) Mean differences (95% CI)
Sex (Boys)	514 (53.6%)	494 (53.2%)	20 (66.7%)	*p *= 0.15	1.76 (0.82, 3.80) *p *= 0.15
Ethnicity (% Caucasian)	895 (95.3%)	867 (95.4%)	28 (93.3%)	* *p *= 0.65	1.47 (0.34, 6.40) *p *= 0.60
Maternal age at birth (years)	30.6 years (30.3–30.9)	30.6 years	31.7 years	*p *= 0.24	Diff −1.09 (−2.91, 0.73)
Gestational age at birth (weeks)	39.9 weeks (39.8–40.0)	39.9 weeks	39.5 weeks	*p *= 0.16	Diff 0.44 (−1.76, 1.05)
Socioeconomic class					
Managerial	402 (61.6%)	392 (61.6%)	10 (58.8%)	* *p *= 0.24	^**^Excluded due to empty cells
Intermediate	151 (23.1%)	144 (22.6%)	7 (41.2%)		
Routine	78 (11.9%)	78 (12.3%)	0 (0%)		
Not working	17 (2.6%)	17 (2.7%)	0 (0%)		
Smoking during pregnancy (Y)					
Maternal	109 (11.5%)	107 (11.7%)	2 (6.7%)	* *p *= 0.56	0.54 (0.13, 2.30)
Paternal	270 (28.6%)	265 (29.0%)	5 (16.7%)	*p *= 0.14	0.49 (0.19, 1.30)
Smoking at child's age 1 year (Y)					
Maternal	129 (13.5%)	125 (13.5%)	4 (13.3%)	* *p *= 1.00	0.98 (0.34, 2.87)
Paternal	250 (26.1%)	245 (26.4%)	5 (16.7%)	*p *= 0.23	0.56 (0.21, 1.47)
Breastfeeding (Y)	660 (71.7%)	640 (71.6%)	20 (74.1%)	*p *= 0.78	1.13 (0.47, 2.71)
Peanut consumption (Y)					
Before pregnancy	88 (91.7%)	62 (92.5%)	26 (89.7%)	* *p *= 0.69	0.70 (0.16, 3.14)
In pregnancy	78 (81.2%)	55 (82.1%)	23 (79.3%)	*p *= 0.75	0.84 (0.28, 2.50)
During breastfeeding (in BF only)	49 (76.6%)	34 (77.3%)	15 (75%)	*p *= 0.84	0.88 (0.26, 3.03)
Pet ownership (Y)					
Cat ownership in pregnancy and early life	193 (20.6%)	183 (20.1%)	10 (33.3%)	***p *= 0.078**	1.98 (0.99, 4.31)
Dog ownership in pregnancy and early life	154 (16.4%)	150 (16.5%)	4 (13.3%)	* *p *= 0.81	0.78 (0.27, 2.26)
Older sibling (Y)	511 (53.6%)	496 (53.7%)	15 (50%)	*p *= 0.69	0.86 (0.42, 1.78)
Day‐care attendance (Y)	629 (70.0%)	610 (69.9%)	19 (73.1%)	*p *= 0.73	1.17 (0.49, 2.82)
Parental atopy (Y)	771 (82.7%)	745 (82.3%)	26 (96.3%)	* *p *= 0.07	5.58 (0.75, 41.4)
Maternal	542 (58.3%)	525 (58.2%)	17 (63.0%)	*p *= 0.62	1.22 (0.55, 2.70)
Paternal	588 (63.8%)	566 (63.2%)	22 (81.5%)	***p *= 0.052**	2.56 (0.96, 6.82)
Parental asthma (Y)	291 (30.3%)	278 (29.9%)	13 (43.3%)	*p *= 0.12	1.79 (0.86, 3.74)
Maternal	187 (19.5%)	179 (19.3%)	8 (26.7%)	*p *= 0.31	1.52 (0.67, 3.48)
Paternal	137 (14.3%)	131 (14.1%)	6 (20%)	*p *= 0.37	1.52 (0.61, 3.79)
Eczema in the first years of life (Y)	318 (35.1%)	298 (33.9%)	20 (74.1%)	***p *< 0.001**	5.56 (2.33, 13.3)
Eczema scoring at age 1 year					
No eczema	344 (82.7%)	340 (84.4%)	4 (30.8%)		Ref Variable
Mild	54 (13.0%)	50 (12.4%)	4 (30.8%)		6.8 (1.65, 28.1)
Mod‐severe	18 (4.3%)	13 (3.2%)	5 (30.5%)		32.7 (7.8, 136.0)
*FLG* loss‐of‐function mutations (Caucasian only)					
Any of the 6 mutations	79 (10.7%)	74 (10.4%)	5 (20%)	*p *= 0.13	2.16 (0.79, 5.92)
Allergic sensitization (SPT)					
Sensitized (any allergen), age 1 year	46 (11.2%)	37 (9.3%)	9 (69.2%)	* ***p *< 0.001**	21.9 (6.43, 74.6)
Sensitized (any allergen), age 3 years	190 (22.8%)	172 (21.3%)	18 (72%)	***p *< 0.001**	9.49 (3.90, 23.1)
Egg sensitization (by SPT/sIgE)					
Sensitized, age 1 year	43 (10.6%)	34 (8.7%)	9 (69.2%)	* ***p *< 0.001**	23.7 (6,93, 81.0)
Sensitized, age 3 years	46 (5.7%)	38 (4.9%)	8 (33.3%)	***p *< 0.001**	9.79 (3.94, 24.3)
Parentally reported wheeze					
In the first year of life	251 (27.4%)	241 (27.1%)	10 (37.0%)	*p *= 0.26	1.58 (0.71, 3.50)
Parentally reported rhinitis					
In the first 3 year of life	39 (4.3%)	36 (4.1%)	3 (11.1%)	* *p *= 0.11	2.92 (0.84, 10.1)

* Denotes when *p*‐value is provided by Fisher's exact test, Bold font denotes *p*‐values with statistical (or near statistical) significance, Ref Variable = reference variable.

#### 3.2.1 Multivariate analysis

We performed multivariate analyses accounting for gender, cat ownership, parental atopy, eczema in infancy and egg sensitization at age 3 years; results are shown in Table 2. Eczema in the first year of life (OR = 4.4, 95% CI 1.5–13.2, *p* = 0.007) and egg sensitization at age 3 years (OR = 9.7, 95% CI 3.3–28.9, *p *< 0.001) were independent associates of peanut allergy. Cat ownership during pregnancy/early life was also associated with an increased risk (OR = 3.0, 95% CI 1.1–8.4, *p* = 0.04).

**TABLE 2:** Multivariate logistic regression analysis models of associates for peanut allergy

Variables	Model 1			Model 2		
	aOR	95% CI	*p*‐Value	aOR	95% CI	*p*‐Value
Sex (Male)	1.64	0.61–4.43	0.32	1.66	0.67–4.11	0.27
Cat ownership in pregnancy and early life	2.96	1.05–8.35	0.04	2.83	1.11–7.21	0.03
Parental atopy	2.78	0.33–23.4	0.35	3.23	0.41–25.1	0.26
Eczema in the first year of life	4.43	1.49–13.2	0.007	4.03	1.51–10.7	0.005
Egg sensitization at age 3 years	9.71	3.27–28.9	<0.001	6.54	2.36–18.1	<0.001
*FLG* loss‐of‐function mutations	1.35	0.42–4.33	0.61	n/a	n/a	n/a

Note

Model 1: FLG loss‐of‐function mutations included.

Model 2: FLG loss‐of‐function mutations excluded.

We also performed multivariate models which included maternal peanut consumption during breastfeeding (among breastfed infants) (Table S3). The only significant associate of peanut allergy in this model was early‐life eczema.

### 3.3 FLG loss‐of‐function mutations and peanut allergy in children with and without eczema

We further explored the association between *FLG* mutations, eczema and peanut allergy among 700 Caucasian participants with complete data by stratifying the population according to whether the child had eczema in the first year of life. Among 238 children with early‐life eczema, there was no difference in the frequency of *FLG* mutations carriers between peanut‐allergic children and those not allergic (16.7% vs. 19.1%, *p* = 1.00) (Table 3). In contrast, among 462 children without eczema, those who carried *FLG* mutations were almost 8 times more likely to have peanut allergy compared to those with *FLG* wild‐type (OR 7.9, 95% CI 1.4–45.3, *p *= 0.02). However, since peanut allergy was rare in children without early‐life eczema (6/462), these results need to be interpreted with caution.

**TABLE 3:** Peanut allergy and *FLG* loss‐of‐function mutations, stratified by the presence of eczema in the first year of life; analysis among children with White European ancestry

Variables: Categorical (proportions, %)	Study population (*n* = 700)	No peanut allergy (*n* = 676)	Peanut allergy (*n* = 24)	*p*‐Value	Odds ratio (95% CI) *p*‐value
Eczema in the first year of life *FLG* Mutations (*n* = 238)					
No (%)	193 (81.1%)	178 (80.9%)	15 (83.3%)	* *p *= 1.00	0.85 (0.23, 3.06)
Yes (%)	45 (18.9%)	42 (19.1%)	3 (16.7%)		*p *= 0.80
No Eczema in the first year of life *FLG* Mutations (*n* = 462)					
No (%)	433 (93.7%)	429 (94.1%)	4 (66.7%)	* ***p *= 0.049**	7.94 (1.39, 45.3)
Yes (%)	29 (6.3%)	27 (5.9%)	2 (33.3%)		***p *= 0.020**

* Denotes when *p*‐value is provided by Fisher's exact test, Bold font denotes *p*‐values with statistical (or near statistical) significance.

### 3.4 Longitudinal profiles of sensitization and allergic diseases in children with peanut allergy

There was a steady rise in the point prevalence of allergic sensitization among peanut‐allergic children and those not allergic to peanut over time (Figure S1), with the prevalence of sensitization being consistently and significantly higher in peanut‐allergic children at all time‐points (Figure S1a). Despite the overall increase in sensitization, there was a gradual reduction in the proportion of children sensitized to egg, and the association of egg sensitization with peanut allergy weakened substantially with increasing age (Figure S1b). In contrast, associations of sensitization to inhalant allergens with peanut allergy strengthened with increasing age (Figure S1c–f).

We observed significantly higher rates of eczema to age of 8 years (Figure S2a), and rhinitis and wheeze throughout childhood (Figures S2, S3), in peanut‐allergic children compared to those not allergic to peanut. Peanut‐allergic children were more likely to have unscheduled visit to GPs and emergency departments with wheeze (Figure S3bc) and to have a doctor‐diagnosed asthma and receive asthma medication (Figure S4).

### 3.5 Association between peanut allergy and data‐driven phenotypes

We previously described longitudinal sensitization clusters derived using machine learning.^33^ Using this classification, peanut allergy was associated with the multiple early and multiple late sensitization, but not to other sensitization clusters (Table 4). This association was strikingly strong for multiple early sensitization (OR 59.1, 95% CI 13.4–260).

**TABLE 4:** Association of peanut allergy with different wheeze phenotypes^32^ and longitudinal trajectories of sensitization and developmental profiles of allergic diseases determined using data‐driven methods

Variable Categorical (proportions, %) numerical (mean, SD)	Study population (*n* = 959)	No peanut allergy (*n* = 929)	Peanut allergy (N = 30)	Odds ratio (95% CI)	*p*‐Value
Wheeze phenotypes^32^					
Never wheezed	410 (43.3%)	404 (44.1%)	6 (20%)	Ref Variable	Ref Var
Transient wheeze	239 (25.2%)	232 (25.3%)	7 (23.3%)	2.03 (0.67, 6.12)	*p *= 0.21
Intermittent wheeze	114 (12.0%)	112 (12.8%)	2 (6.7%)	1.20 (0.24, 6.04)	*p *= 0.82
Late‐onset wheeze	55 (5.8%)	52 (12.8%)	3 (10%)	3.88 (0.94, 16.0)	*p *= 0.06
Persistent wheeze	129 (13.6%)	117 (12.8%)	12 (40%)	6.91 (2.54, 18.8)	*p *< 0.001
Clusters of allergic sensitization^33^					
1. No atopic vulnerability	468 (55.2%)	466 (56.7%)	2 (7.7%)	Ref Variable	Ref Var
2. Predominantly non‐HDM	97 (11.4%)	97 (11.8%)	0 (0%)	Omitted as empty	OMITTED
3. Predominantly HDM	47 (5.5%)	47 (5.7%)	0 (0%)	Omitted as empty	OMITTED
4. Multiple late	147 (17.3%)	141 (17.2%)	6 (23.1%)	9.91 (1.98, 49.7)	*p *= 0.005
5. Multiple early	89 (10.5%)	71 (8.6%)	18 (69.2%)	59.1 (13.4, 260)	*p *< 0.001
Developmental profiles of allergic diseases^34^					
1. No disease	369 (38.5%)	366 (39.4%)	3 (10.0%)	Ref Variable	Ref Var
2. Persistent eczema & Late‐onset Rhinitis	72 (7.5%)	65 (7.0%)	7 (23.3%)	13.1 (3.31, 52.1)	*p *< 0.001
3. Eczema only	156 (16.3%)	155 (16.7%)	1 (3.3%)	0.79 (0.08, 7.63)	*p *= 0.84
4. Transient wheeze	62 (6.5%)	60 (6.5%)	2 (6.7%)	4.07 (0.67, 7.63)	*p *= 0.13
5. Persistent wheeze & late‐onset rhinitis	84 (8.8%)	82 (8.8%)	2 (6.7%)	2.98 (0.49, 18.1)	*p *= 0.24
6. Persistent eczema & wheeze	48 (5.0%)	45 (4.9%)	3 (10.0%)	8.13 (1.59, 41.5)	*p *= 0.012
7. Rhinitis only	114 (11.9%)	112 (12.1%)	2 (6.7%)	2.18 (0.36, 13.2)	*p *= 0.40
8. Atopic March	54 (5.6%)	44 (4.7%)	10 (33.3%)	27.7 (7.35, 104.6)	*p *< 0.001

Abbreviation: HDM, house dust mite; Ref Variable, reference variable.

Differences were also noted in relation to wheeze phenotypes,^32^ in that peanut allergy was strongly associated with persistent wheeze (OR 6.9, 95% CI 2.5–18.8, *p *< 0.001), but not any other wheeze phenotype (Table 4).

Associations also differed for different developmental profiles of allergic diseases.^34^ Peanut allergy was strongly associated with the multimorbidity phenotype (eczema + wheeze + rhinitis, OR = 27.7, 95% CI 7.35–104.6, *p *< 0.001). There was also an association with other comorbidity clusters (persistent eczema and rhinitis, OR = 13.1, 95% 3.3–52.1, *p *< 0.001; persistent eczema and wheeze, OR = 8.1, 95% CI 1.6–41.5, *p *= 0.012), but not with the eczema only class (Table 4).

### 3.6 Peanut allergy and asthma severity in school age

Peanut‐allergic children were more likely to have asthma diagnosis at age 11 years (OR 4.0, 95% CI 1.9–8.5, *p *< 0.001) (Table S4). Association between peanut allergy and asthma severity is shown in Table 5. Among asthmatics, those with peanut allergy were numerically more likely to receive oral corticosteroid prescription, have severe exacerbations and hospital admissions for asthma exacerbation, but none of these reached statistical significance. Peanut‐allergic asthmatics were three times more likely to have multiple (≥4) exacerbations compared to those not allergic to peanut (OR = 2.9, 95% CI 0.9–9.3, *p *= 0.07), but this difference was not statistically significant.

**TABLE 5:** Asthma severity among asthmatics with and without peanut allergy

Variable Categorical (%) Numerical (mean, SD)	All	Children with asthma		*p*‐value	Odds ratio (95% CI)
		No peanut allergy	Peanut allergy		
Oral corticosteroid prescription ever	*n* = 144 56 (38.9%)	*n* = 132 50 (37.9%)	*n* = 12 6 (50%)	*p *= 0.41	1.64 (0.50, 5.36)
Hospital admissions with wheeze	*n* = 144 38 (26.4%)	*n* = 132 33 (25%)	*n* = 12 5 (41.7%)	*p *= 0.21	2.14 (0.64, 7.21)
Severe exacerbations (≥1)	*n* = 176 96 (54.6%)	*n* = 159 84 (52.8%)	*n* = 17 12 (70.6%)	*p *= 0.16	2.14 (0.72, 6.37)
Severe exacerbations in the first 8 years of life	*n* = 176	*n* = 159	*n* = 17		
0	80 (45.5%)	75 (47.2%)	5 (29.4%)	* *p *= 0.34	Reference variable
1	23 (13.1%)	21 (13.2%)	2 (11.8%)		1.43 (0.26, 7.90)
2	10 (5.7%)	9 (5.7%)	1 (5.9%)		1.67 (0.17, 15.9)
3	8 (4.6%)	8 (5.0%)	0 (0%)		Omitted as empty
≥4	55 (31.3%)	46 (28.9%)	9 (52.9%)		2.93 (0.93, 9.30)

* Denotes when *p*‐value is provided by Fisher's exact test, Ref Variable = reference variable.

## 4 DISCUSSION

In a population‐based birth cohort which included food challenges to diagnose peanut allergy, we confirmed that eczema in the first year of life and egg sensitization in the first three years are associated with peanut allergy in school age. Among children with eczema, there was no association between peanut allergy and *FLG* genotype. In contrast, among those without eczema, carriers of *FLG* loss‐of‐function mutations were almost 8 times more likely to have peanut allergy compared to children without *FLG* mutations. However, given the low frequency of peanut allergy in children without infantile eczema, caution is needed when interpreting these results.

Cat ownership in pregnancy and early life was associated with almost threefold increased risk of peanut allergy. Peanut allergy was markedly more common among children with persistent wheezing, but not any other wheeze phenotype, with approximately sevenfold increase in risk. Also of note is a near‐exclusive association between peanut allergy and multiple allergic sensitization classes (both early and late^33^), with almost 60‐fold increase in risk among individuals assigned to multiple early sensitization. When we investigated the association between peanut allergy and machine‐learning derived developmental profiles of eczema, wheeze and rhinitis,^34^ we observed consistent association for all profiles characterized by persistent eczema and multimorbidity, but there was no association with class characterized by eczema only, with no atopic comorbidities. Finally, although peanut‐allergic children were more likely to have asthma diagnosis, among children with asthma, there was no convincing evidence of a strong association between peanut allergy and asthma severity.

A key limitation of our study is relatively small number of peanut‐allergic children available for the analysis, but this is inevitable when studying a relatively uncommon outcome in an unselected birth cohort. Therefore, our results need to be interpreted with caution. To increase the size of peanut‐allergic group, we recruited 12 children from a tertiary referral allergy clinic with confirmed peanut allergy who had longitudinal data to enable our analyses; exclusion of these children did not materially alter our findings (data available on request).

We acknowledge that not all children classified as peanut allergic underwent OFC. Four participants who declined OFC (*n* = 2) or had equivocal OFC (*n* = 2) were classified as peanut allergic based on clinical information and Ara‐h‐2 sIgE consistent with allergy.^29^

Another limitation of our study is that the population is not ethnically diverse, and the results are therefore not directly transferable to other ethnic groups. Furthermore, we could not ascertain with certainty which of the associates of peanut allergy are shared risk factors for atopy/sensitization in general. To address this important question, we need large case–control studies comparing patients with OFC‐confirmed peanut allergy with individuals who are peanut‐sensitized but tolerant (who in our analysis outnumber those with true peanut allergy by the factor 3:1). Such studies are likely to require multi‐cohort and multi‐study collaborations to ensure adequate sample size.

One of the strengths is that our birth cohort incorporated OFC to diagnose peanut allergy. Most studies to date relied on clinical history and/or IgE sensitization to define for food allergy, which may overestimate prevalence.^45^ Another strength is the unselected design, with longitudinal phenotyping from birth and a high follow‐up rate, thereby minimizing recall bias. Our data will be valuable for comparison with population‐based studies in children with objective food allergy outcomes (e.g. HealthNuts).^46^

Importantly, our previous work allowed longitudinal phenotyping of participants into data‐driven clusters of sensitization, wheeze and allergic multimorbidity,^32^, ^33^, ^34^ thereby enabling us to gain invaluable insight into the relationship between peanut allergy and developmental trajectories of atopic diseases. A strong association between early‐life eczema and development of food allergy is well recognized, with the risk being higher in children with earlier onset and more severe skin disease.^26^, ^47^ We have extended these findings to demonstrate that peanut allergy is associated with all developmental patterns of childhood allergic diseases which include persistent eczema with allergic comorbidities (persistent wheeze and rhinitis), but there was no association with eczema or wheeze presenting as single diseases (particularly transient). We observed strong associations between peanut allergy and atopic multimorbidity and multiple sensitization clusters. We acknowledge that these observation do not imply any specific causal relationships and that the results are predominantly hypothesis‐generating.

Our findings provide further evidence for the importance of a disrupted skin barrier in early life for the development of peanut allergy. We have shown that among children with eczema (and thereby disrupted skin barrier), *FLG* mutations did not further increase the risk. In contrast, in those without eczema, *FLG* mutations (which result in skin barrier dysfunction^48^) were associated with almost eightfold increased risk. These findings are consistent with the dual allergen exposure hypothesis,^27^ which describes the paradigm where low‐dose allergen exposure through an impaired skin leads to development of sensitization and allergy, whereas a higher‐dose oral exposure and antigen presentation to the gut immune system induces immune tolerance.

Sharing a home with a cat during pregnancy and early life was associated with a threefold increase in the risk of subsequent peanut allergy. We propose that this effect may be mediated via the development of early inhalant sensitization, thereby creating a permissive milieu for allergic multimorbidities.^49^ This is consistent with our current findings that cat sensitization was the most common inhalant sensitization among peanut‐allergic children at age 1 year (and the only early‐life inhalant allergen sensitization which differed significantly between peanut‐allergic and non‐allergic children), and our previous findings that early‐life cat ownership increases the risk of cat sensitization by age 1 (but not thereafter)^50^ and that this effect is particularly strong among children with *FLG* mutations.^42^

It has been proposed that asthmatic children with peanut allergy have more severe asthma than asthmatics without peanut allergy.^13^ However, although our analysis revealed that peanut‐allergic children were more likely to have a diagnosis of asthma, we found no consistent evidence that among asthmatic children, peanut allergy was associated with any markers of increased asthma severity. However, this warrants further investigation in larger studies.

In conclusion, our data suggest that peanut allergy is strongly associated with atopic disease trajectories characterized by early‐onset and persistent eczema and wheeze, but not with transient eczema or wheeze. Among children without eczema, carriers of *FLG* loss‐of‐function mutations were more likely to have peanut allergy, but in those with eczema, there was no association between peanut allergy and *FLG* genotype.

## CONFLICT OF INTEREST

Dr. Custovic reports personal fees from Novartis, personal fees from Regeneron / Sanofi, personal fees from Thermo Fisher Scientific, personal fees from Boehringer Ingelheim, personal fees from Novartis and personal fees from Philips, outside the submitted work. AS reports lecture fees from Thermo Fisher Scientific. Other authors have no competing interests to declare.

## AUTHOR CONTRIBUTIONS

AC, CK, AS and CSM conceived and planned the study; NN and GK completed all OFCs; CK and SH analysed the data; CK, AC and PJT wrote the manuscript. All authors provided critical feedback and helped shape the research, analysis and manuscript.

## Supporting information

Supplementary Material

## References

[1] Hourihane JO , Aiken R , Briggs R , et al. The impact of government advice to pregnant mothers regarding peanut avoidance on the prevalence of peanut allergy in United Kingdom children at school entry. J Allergy Clin Immunol. 2007;119(5):1197‐1202. 17353036 10.1016/j.jaci.2006.12.670

[2] Nicolaou N , Poorafshar M , Murray C , et al. Allergy or tolerance in children sensitized to peanut: prevalence and differentiation using component‐resolved diagnostics. J Allergy Clin Immunol. 2010;125(1):191‐197.e1‐13. 20109746 10.1016/j.jaci.2009.10.008

[3] Venter C , Hasan Arshad S , Grundy J , et al. Time trends in the prevalence of peanut allergy: three cohorts of children from the same geographical location in the UK. Allergy. 2010;65(1):103‐108. 20078504 10.1111/j.1398-9995.2009.02176.x

[4] Bunyavanich S , Rifas‐Shiman SL , Platts‐Mills TA , et al. Peanut allergy prevalence among school‐age children in a US cohort not selected for any disease. J Allergy Clin Immunol. 2014;134(3):753‐755. 25086866 10.1016/j.jaci.2014.05.050 PMC4149917

[5] Sicherer SH , Sampson HA . Food allergy: A review and update on epidemiology, pathogenesis, diagnosis, prevention, and management. J Allergy Clin Immunol. 2018;141(1):41‐58. 29157945 10.1016/j.jaci.2017.11.003

[6] Peters RL , Allen KJ , Dharmage SC , et al. Natural history of peanut allergy and predictors of resolution in the first 4 years of life: A population‐based assessment. J Allergy Clin Immunol. 2015;135(5):1257‐1266.e1‐2. 25725989 10.1016/j.jaci.2015.01.002

[7] Sicherer SH , Muñoz‐Furlong A , Godbold JH , Sampson HA . US prevalence of self‐reported peanut, tree nut, and sesame allergy: 11‐year follow‐up. J Allergy Clin Immunol. 2010;125(6):1322‐1326. 20462634 10.1016/j.jaci.2010.03.029

[8] Kotz D , Simpson CR , Sheikh A . Incidence, prevalence, and trends of general practitioner‐recorded diagnosis of peanut allergy in England, 2001 to 2005. J Allergy Clin Immunol. 2011;127(3):623‐630.e1. 21236479 10.1016/j.jaci.2010.11.021

[9] Bock SA , Muñoz‐Furlong A , Sampson HA . Further fatalities caused by anaphylactic reactions to food, 2001–2006. J Allergy Clin Immunol. 2007;119(4):1016‐1018. 17306354 10.1016/j.jaci.2006.12.622

[10] Baseggio Conrado A , Ierodiakonou D , Gowland MH , Boyle RJ , Turner PJ . Food anaphylaxis in the United Kingdom: analysis of national data, 1998–2018. BMJ. 2021;372:n251. 33597169 10.1136/bmj.n251 PMC7885259

[11] Pumphrey RS , Gowland MH . Further fatal allergic reactions to food in the United Kingdom, 1999–2006. J Allergy Clin Immunol. 2007;119(4):1018‐1019. 17349682 10.1016/j.jaci.2007.01.021

[12] Maris I , Dolle‐Bierke S , Renaudin JM , et al. Peanut‐induced anaphylaxis in children and adolescents: Data from the European Anaphylaxis Registry. Allergy. 2021;76(5):1517‐1527. 33274436 10.1111/all.14683

[13] di Palmo E , Gallucci M , Cipriani F , Bertelli L , Giannetti A , Ricci G . Asthma and food allergy: which risks? Medicina (Kaunas). 2019;55(9):509. 10.3390/medicina55090509 PMC6780261 31438462

[14] Saglani S , Wisnivesky JP , Charokopos A , Pascoe CD , Halayko AJ , Custovic A . Update in asthma 2019. Am J Respir Crit Care Med. 2020;202(2):184‐192. 32338992 10.1164/rccm.202003-0596UP

[15] Stiefel G , Anagnostou K , Boyle RJ , et al. BSACI guideline for the diagnosis and management of peanut and tree nut allergy. Clin Exp Allergy. 2017;47(6):719‐739. 28836701 10.1111/cea.12957

[16] Ford LS , Taylor SL , Pacenza R , Niemann LM , Lambrecht DM , Sicherer SH . Food allergen advisory labeling and product contamination with egg, milk, and peanut. J Allergy Clin Immunol. 2010;126(2):384‐385. 20621349 10.1016/j.jaci.2010.05.034

[17] Cherkaoui S , Ben‐Shoshan M , Alizadehfar R , et al. Accidental exposures to peanut in a large cohort of Canadian children with peanut allergy. Clin Transl Allergy. 2015;5:16. 25861446 10.1186/s13601-015-0055-x PMC4389801

[18] Bollinger ME , Dahlquist LM , Mudd K , Sonntag C , Dillinger L , McKenna K . The impact of food allergy on the daily activities of children and their families. Ann Allergy Asthma Immunol. 2006;96(3):415‐421. 16597075 10.1016/S1081-1206(10)60908-8

[19] Duca B , Patel N , Turner PJ . GRADE‐ing the benefit/risk equation in food immunotherapy. Curr Allergy Asthma Rep. 2019;19(6):30. 31025125 10.1007/s11882-019-0862-6 PMC6483945

[20] Vickery BP , Hourihane JO , Adelman DC . Oral immunotherapy for peanut allergy. N Engl J Med. 2019;380(7):691‐692. 10.1056/NEJMc1817331 30763186

[21] Du Toit G , Sampson HA , Plaut M , Burks AW , Akdis CA , Lack G . Food allergy: Update on prevention and tolerance. J Allergy Clin Immunol. 2018;141(1):30‐40. 29191680 10.1016/j.jaci.2017.11.010 PMC12548800

[22] Fox AT , Kaymakcalan H , Perkin M , du Toit G , Lack G . Changes in peanut allergy prevalence in different ethnic groups in 2 time periods. J Allergy Clin Immunol. 2015;135(2):580‐582. 25441289 10.1016/j.jaci.2014.09.022

[23] Koplin JJ , Peters RL , Ponsonby AL , et al. Increased risk of peanut allergy in infants of Asian‐born parents compared to those of Australian‐born parents. Allergy. 2014;69(12):1639‐1647. 25041549 10.1111/all.12487

[24] Bloomfield SF , Rook GA , Scott EA , Shanahan F , Stanwell‐Smith R , Turner P . Time to abandon the hygiene hypothesis: new perspectives on allergic disease, the human microbiome, infectious disease prevention and the role of targeted hygiene. Perspect Public Health. 2016;136(4):213‐224. 27354505 10.1177/1757913916650225 PMC4966430

[25] Lack G . Update on risk factors for food allergy. J Allergy Clin Immunol. 2012;129(5):1187‐1197. 22464642 10.1016/j.jaci.2012.02.036

[26] Lack G , Fox D , Northstone K , Golding J . Factors associated with the development of peanut allergy in childhood. N Engl J Med. 2003;348(11):977‐985. 12637607 10.1056/NEJMoa013536

[27] Lack G . Epidemiologic risks for food allergy. J Allergy Clin Immunol. 2008;121(6):1331‐1336. 18539191 10.1016/j.jaci.2008.04.032

[28] Brown SJ , Asai Y , Cordell HJ , et al. Loss‐of‐function variants in the filaggrin gene are a significant risk factor for peanut allergy. J Allergy Clin Immunol. 2011;127(3):661‐667. 21377035 10.1016/j.jaci.2011.01.031 PMC3081065

[29] Nicolaou N , Murray C , Belgrave D , Poorafshar M , Simpson A , Custovic A . Quantification of specific IgE to whole peanut extract and peanut components in prediction of peanut allergy. J Allergy Clin Immunol. 2011;127(3):684‐685. 21272928 10.1016/j.jaci.2010.12.012

[30] Brough HA , Simpson A , Makinson K , et al. Peanut allergy: effect of environmental peanut exposure in children with filaggrin loss‐of‐function mutations. J Allergy Clin Immunol. 2014;134(4):867‐875.e1. 25282568 10.1016/j.jaci.2014.08.011 PMC4188983

[31] Nakamura T , Haider S , Fontanella S , Murray CS , Simpson A , Custovic A . Modelling trajectories of parentally reported and physician‐confirmed atopic dermatitis in a birth cohort study. Br J Dermatol. 2021;186(2):274‐284. 34564850 10.1111/bjd.20767

[32] Lowe LA , Simpson A , Woodcock A , et al. Wheeze phenotypes and lung function in preschool children. Am J Respir Crit Care Med. 2005;171(3):231‐237. 15502115 10.1164/rccm.200406-695OC

[33] Simpson A , Tan VY , Winn J , et al. Beyond atopy: multiple patterns of sensitization in relation to asthma in a birth cohort study. Am J Respir Crit Care Med. 2010;181(11):1200‐1206. 20167852 10.1164/rccm.200907-1101OC

[34] Belgrave DC , Granell R , Simpson A , et al. Developmental profiles of eczema, wheeze, and rhinitis: two population‐based birth cohort studies. PLoS Med. 2014;11(10):e1001748. 25335105 10.1371/journal.pmed.1001748 PMC4204810

[35] Custovic A , Simpson A , Woodcock A . Manchester cohort. Pediatr Pulmonol Suppl. 2004;26:12‐13. 15029579 10.1002/ppul.70033

[36] Simpson BM , Custovic A , Simpson A , et al. NAC Manchester Asthma and Allergy Study (NACMAAS): risk factors for asthma and allergic disorders in adults. Clin Exp Allergy. 2001;31(3):391‐399. 11260150 10.1046/j.1365-2222.2001.01050.x

[37] Asher MI , Keil U , Anderson HR , et al. International Study of Asthma and Allergies in Childhood (ISAAC): rationale and methods. Eur Respir J. 1995;8(3):483‐491. 7789502 10.1183/09031936.95.08030483

[38] Ferris BG . Epidemiology standardization project (American Thoracic Society). Am Rev Respir Dis. 1978;118(6 Pt 2):1‐120. 742764

[39] Patel S , Custovic A , Smith JA , Simpson A , Kerry G , Murray CS . Cross‐sectional association of dietary patterns with asthma and atopic sensitization in childhood ‐ in a cohort study. Pediatr Allergy Immunol. 2014;25(6):565‐571. 25201630 10.1111/pai.12276

[40] Howard R , Belgrave D , Papastamoulis P , Simpson A , Rattray M , Custovic A . Evolution of IgE responses to multiple allergen components throughout childhood. J Allergy Clin Immunol. 2018;142(4):1322‐1330. 29428391 10.1016/j.jaci.2017.11.064 PMC6170973

[41] Semic‐Jusufagic A , Belgrave D , Pickles A , et al. Assessing the association of early life antibiotic prescription with asthma exacerbations, impaired antiviral immunity, and genetic variants in 17q21: a population‐based birth cohort study. Lancet Respir Med. 2014;2(8):621‐630. 24835835 10.1016/S2213-2600(14)70096-7

[42] Simpson A , Brough HA , Haider S , Belgrave D , Murray CS , Custovic A . Early‐life inhalant allergen exposure, filaggrin genotype, and the development of sensitization from infancy to adolescence. J Allergy Clin Immunol. 2020;145(3):993‐1001. 31629803 10.1016/j.jaci.2019.08.041 PMC7057264

[43] Lazic N , Roberts G , Custovic A , et al. Multiple atopy phenotypes and their associations with asthma: similar findings from two birth cohorts. Allergy. 2013;68(6):764‐770. 23621120 10.1111/all.12134

[44] Clark H , Granell R , Curtin JA , et al. Differential associations of allergic disease genetic variants with developmental profiles of eczema, wheeze and rhinitis. Clin Exp Allergy. 2019;49(11):1475‐1486. 31441980 10.1111/cea.13485 PMC6899469

[45] Rona RJ , Keil T , Summers C , et al. The prevalence of food allergy: a meta‐analysis. J Allergy Clin Immunol. 2007;120(3):638‐646. 17628647 10.1016/j.jaci.2007.05.026

[46] Osborne NJ , Koplin JJ , Martin PE , et al. Prevalence of challenge‐proven IgE‐mediated food allergy using population‐based sampling and predetermined challenge criteria in infants. J Allergy Clin Immunol. 2011;127(3):668‐676.e1‐2. 21377036 10.1016/j.jaci.2011.01.039

[47] Martin PE , Eckert JK , Koplin JJ , et al. Which infants with eczema are at risk of food allergy? Results from a population‐based cohort. Clin Exp Allergy. 2015;45(1):255‐264. 25210971 10.1111/cea.12406

[48] Flohr C , England K , Radulovic S , et al. Filaggrin loss‐of‐function mutations are associated with early‐onset eczema, eczema severity and transepidermal water loss at 3 months of age. Br J Dermatol. 2010;163(6):1333‐1336. 21137118 10.1111/j.1365-2133.2010.10068.x

[49] Custovic A , Custovic D , Kljaic Bukvic B , Fontanella S , Haider S . Atopic phenotypes and their implication in the atopic march. Expert Rev Clin Immunol. 2020;16(9):873‐881. 32856959 10.1080/1744666X.2020.1816825

[50] Ihuoma H , Belgrave DC , Murray CS , Foden P , Simpson A , Custovic A . Cat ownership, cat allergen exposure, and trajectories of sensitization and asthma throughout childhood. J Allergy Clin Immunol. 2018;141(2):820‐822 e7. 29111216 10.1016/j.jaci.2017.09.030 PMC5792051

