# Supplementary material for: Early‐life predictors and risk factors of peanut allergy, and its association with asthma in later‐life: Population‐based birth cohort study
Source: Clin Exp Allergy. 2022 Feb 10;52(5):646–57. doi: 10.1111/cea.14103 (PMC9303430; doi:10.1111/cea.14103)
Supplement: Supplementary file 1 — Supplementary Material [file CEA-52-646-s001.docx]

**Early-life predictors and risk factors of peanut allergy, and its association with asthma in later-life: Population-based birth cohort study**

Constantinos Kotsapas, Nicolaos Nicolaou, Sadia Haider, Gina Kerry, Paul Turner, Clare S Murray, Angela Simpson, Adnan Custovic

**Supplementary Appendix**

**Table S1.** List of previous studies which investigated the early-life predictors and risk factors for food and peanut sensitization and allergy.

PA=peanut allergy; SPT=skin prick testing; AD=atopic dermatitis; OFC=oral food challenge; DBPCFC=double blinded placebo-controlled food challenge; FA=food allergy; FS=food sensitisation; RF=risk factors; TEWL=trans-epidermal water loss

| Author  Publication year | Type of Study | Methodology/Background | Relevant finding(s) |
| --- | --- | --- | --- |
| Tariq et al  1996 (1) | Birth cohort | Children born in the Isle of Wight between 1989-1990 and recruited in the IoW birth cohort assessed for PA at age 4y using questionnaires and SPT. | Association of PA with egg allergy (OR 9.9) and eczema (OR 7.3). |
| Ewan P W  1996 (2) | Case Series | 62 patients with PA seen in allergy clinic in Addenbrookes between 1993-1994. | Atopy observed in 53/55 children; all 53 had other allergic disease (asthma, rhinitis, eczema). |
| Hourihane et al  1996 (3) | Cross-sectional study | Patients with suspected PA referred by GP, anaphylaxis campaign or via self-contact, were asked to fill in questionnaires re: other allergic disease in them and in their siblings and two preceding generations. | 7-fold increased risk of peanut allergy if a sibling has peanut allergy. All forms of allergy more common in successive generations, and more common in maternal than paternal relatives. |
| Eigenmann et al  1998 (4) | Case Series | Children presenting to paediatric dermatology clinic at John Hopkins were recruited based on their history of AD. They were assessed for eczema severity and for sensitization and FA using serum sIgE and OFC where indicated. | Approximately one third of children with refractory, moderate-severe AD had IgE-mediated clinical reactivity to food proteins. |
| Sicherer et al  2000 (5) | Twin Study | Twin pairs with at least one member with peanut allergy were recruited through the Food Allergy Network and interviewed via telephone using validated questionnaires. | Significantly higher rate of peanut allergy amongst monozygotic twins (64.3%) than dizygotic twins (6.8%). |
| Lack et al  2003 (6) | Birth cohort | Children born between 1991-1992 in the ALSPAC birth cohort with a reported history of previous reactions to or avoidance of peanut, underwent SPT and DBPCFC for confirmation. | Association of PA with eczematous dermatitis in first 6 months of life and with use of skin preparations containing peanut oils. |
| Hill et al  2007 (7) | EPAAC screening study | Analysis of the screening data for the EPAAC RCT study designed to test the effect of levocetirizine on onset of asthma. Children were assessed for high risk IgE food sensitization to milk, egg and/or peanut as a marker for FA. | Children at high risk of IgE food sensitization (HR-IgE-FS) had the most severe eczema and the youngest age of onset. 64% of infants with severe eczema <3months had HR-IgE FS. |
| Du Toit et al  2008 (8) | Case-control study | Comparison of the prevalence of PA between Jewish schoolchildren and peanut consumption and weaning between Jewish infants, in London and Israel, using a validated questionnaire. | Higher rates of PA in Jewish schoolchildren in the UK. Peanut was introduced earlier and eaten more frequently and in larger quantities amongst Jewish infants in Israel. |
| Fox et al  2009 (9) | Case-control study | Questionnaires to assess the effect of environmental household exposure to peanut to the development of PA in infants up to the age of 12months. Compared 133 children with PA against 160 egg allergic but not PA controls, and another 160 non-atopic controls. | No effect of maternal peanut consumption during pregnancy or lactation; high levels of environmental exposure to peanut during infancy appear to promote sensitization,  The risk higher with peanut butter compared to other forms of peanut. |
| Sicherer et al  2010 (10) | CoFAR birth cohort | Observational study of infants at high risk of developing PA enrolled from 5 sites in the US assessing for RF for peanut sensitization and PA; sIgE >5kUA/l was considered likely indicative of PA. | Frequent consumption of peanut in pregnancy, male sex, non-white race, and presence of IgE to milk and egg associated with peanut sIgE>5kUA/l. |
| Brown et al  2011 (11) | Multi-centre  case-control study | Multi-centre study including patients with OFC-confirmed PA and 1000 controls not sensitised to peanut from ALSPAC, looking at the association of *FLG* mutations and eczema with PA. Results replicated in a Canadian study. | Association of *FLG* loss-of-function mutation with OFC-confirmed PA; association remained significant after controlling for presence of co-existent AD. |
| Tan et al  2012 (12) | Population-based cohort: HealthNuts | HealthNuts (population-based cohort of 12-month infants recruited at immunisations clinics in Melbourne Australia), to investigate the association between *FLG* loss-of-function mutations and development of food sensitization and OFC-confirmed FA. | After adjusting for eczema, *FLG* mutations were associated with food sensitization, with a similar trend for FA. No difference in *FLG* mutations between food-sensitized children with FA and food-sensitized but tolerant children. |
| Du Toit et al  2013 (13) | Observational study: LEAP screening study | Single centre prospective study of infants at high risk for PA who were recruited for the LEAP RCT, looking at appropriate predictors for presence of peanut sensitization on SPT and sIgE. | The most important predictors of peanut sensitization were presence of egg allergy and eczema, with eczema of increasing severity and duration associated with an increasing risk for sensitization. Black race associated with higher risk for peanut sensitisation on sIgE, but lower risk on SPT. |
| Koplin et al  2013 (14) | Population-based cohort: HealthNuts | HealthNuts study: relationship between family history of allergic disease and OFC-confirmed FA in 12-month-old infants. | Children with one immediate family member with allergic disease history had a modest increase in FA. Having 2 or more family members with history of allergic disease strongly predictive of FA. Maternal history more strongly associated with FA. Maternal and paternal history of asthma and rhinitis were the only predictors for PA in infants. |
| Koplin et al  2014 (15) | Population-based cohort: HealthNuts | HealthNuts study: The relationship between parental country of birth. | Children with parents born in East Asia (but not those with parents born in UK or Europe) were >3 times more likely to have PA than infants with Australian-born parents. |
| Flohr et al  2014 (16) | Case series study | 619 exclusively BF infants from the general population recruited into the EAT interventional study were assessed before randomization for eczema and eczema severity and its relation to food sensitization, *FLG* loss-of-function mutation carriage and TEWL. | Children with AD were more likely to be sensitised; this effect was stronger with increasing eczema severity, but it was independent of *FLG* loss-of-function mutation carriage, TEWL and AD phenotype (flexural vs non-flexural). Concluded that AD is the main skin-related factor for food sensitization. |
| Brough et al  2014 (17) | MAAS birth cohort | Relationship of environmental peanut exposure objectively assessed in the dust samples from homes in early life with *FLG* loss-of-function mutations and OFC-confirmed PA at age 8y. | A dose-response relationship between early-life domestic peanut allergen levels and subsequent development of OFC-confirmed PA, but only in children with a *FLG* loss-of-function mutation. |
| Du Toit et al  2015 (18) | RCT; LEAP trial | 4-11-month-old infants at high risk of peanut allergy were randomized to either early peanut introduction or avoidance and assessed for development of PA at 60months. | There was a relative risk reduction in the development of PA in the early consumption group of 86% in those with negative SPT and 70% in those with positive SPT at enrolment. |
| Martin et al  2015 (19) | Population-based cohort: HealthNuts | HealthNuts: Relationship between eczema and OFC-confirmed FA. | Eczema of all clinical severity strongly associated with IgE mediated FA. |
| Panjari et al  2016 (20) | Cross-sectional study | Using the 2010 school entrant questionnaire authors assessed parentally-reported PA in 5y old children entering school in Victoria, Australia. | PA was more commonly reported amongst children with mothers with higher socioeconomic status index. Prevalence appeared less in regional Victoria compared to Melbourne. |
| Grimshaw et al  2016 (21) | PIFA birth cohort | UK birth cohort part of the EuroPrevall project that included 1140 babies born between 2006-08. Looked at infants with suspected (IgE and non-IgE) FA based on questionnaires, sensitization testing, and DBPCFC if indicated. | Presence of eczema, rhinitis, and a healthy dietary pattern score were predictors for IgE-mediated FA. |
| Perkin et al  2016 (22) | RCT; EAT trial | 3m exclusively BF infants recruited from the general population in England and Wales were randomized to either early dietary introduction of 6 allergenic foods or standard weaning practice and assessed for development of FA and eczema at 3y. | Diagnosis of (any) FA was associated with presence of eczema at enrolment and also non-white race and having siblings. |
| Perkin et al  2021 (23) | Post-hoc analysis of data from EAT trial | Retrospectively analysed data obtained in the EAT interventional study, to assess the relation between moisturization, eczema and dry skin, and the development of FA. | A dose-response relationship between frequency of moisturization at 3 months and subsequent development of FA. *FLG* mutation carrier infants with no visible eczema at 3-months were likely to have increased frequency of moisturization. |

**METHODS**

### **The Manchester Asthma and Allergy Study (MAAS)**

MAAS is an unselected (i.e. population-based) birth cohort study established in 1995 in Manchester, UK (24). The study participants were recruited from a mixed urban-rural population residing within 50 square miles of South Manchester and Cheshire in the maternity catchment area of Wythenshawe and Stepping Hill Hospitals. All pregnant women were screened for eligibility at antenatal visits (8-10^th^ week of pregnancy). Participants were recruited prenatally between October 1995 and July 1997. Pregnant women and their partners completed questionnaires about environmental exposures and the history of allergic diseases, and had skin prick testing (SPT) to four common aeroallergens (house dust mite-HDM, cat, dog, and mixed grasses).

Of the 1499 couples who met the inclusion criteria (≤10 weeks of pregnancy, maternal age ≥18 years, and questionnaire and skin prick data test available for both parents), 288 declined to take part in the study and 27 were lost to follow-up between recruitment and the birth of a child. A total of 1184 children were born into the study between February 1996 and April 1998. They were followed prospectively and attended follow-up clinics for assessments, which included administration of validated questionnaires, lung function measurements, skin prick testing, and the collection of biological samples (serum, plasma and urine). The study was approved by the North West – Greater Manchester East Research Ethics Committee.

Children were followed prospectively, and attended review clinics at ages 1, 3, 5, 8 and 11years. At age 1 year, only children with either both atopic parents, or no atopic parents who lived in homes without a pet were invited to attend clinical follow up. At all other time points for all other measures all children were invited to participate. At each visit, structured validated interviewer-administered questionnaires, based on the American Thoracic Society (ATS) (25) and/or the International Study of Asthma and Allergies in Childhood (ISAAC) questionnaire format (26, 27) were used to collect information for environmental exposures, parentally reported symptoms, physician-diagnosed allergic diseases, and any medication or treatments received.

*Allergic sensitization*. Sensitization was ascertained by skin prick tests (SPT) at all ages for 7 allergens (*Dermatophagoides pteronyssinus*, cat, dog, grass pollen, moulds, milk, and egg [Bayer, Elkahrt, Ind, US]). From age 8 years, SPTs were additionally performed for tree pollen (birch) and peanut (total of 9 allergens tests).

**Data from primary care healthcare records**

We extracted data from General Practitioner (GP)-held medical records including prescriptions, acute episodes, medication prescriptions and hospital admissions. A trained physician reviewed the written and computerized primary care medical records for each child. All consultations with health care providers including hospital admissions, hospital outpatient visits and use of the out of hours services, with linked prescriptions (drug name, route of administration and the dose) was separately entered by the date of the event, allowing the calculation of child’s age in days at each event. Information captured included location, the type of visit, reason for that particular consultation and any relevant symptoms and diagnoses (such as LRTI or bronchiolitis).

**Oral Food Challenge to ascertain peanut allergy (28)**

*Inclusion criteria for open challenge.*

• Positive SPT and/or detectable peanut-specific IgE, eating peanuts (freely)

*Inclusion criteria DBPCFC*

• Positive SPT and detectable sIgE (>0.35 kUa/L) with or without a history of reaction

• Positive SPT, no sIgE available, no history of reaction for more than 2 years

• Positive SPT, no sIgE available, does not eat nuts

• Detectable sIgE (>0.35 kUa/L), no SPT available, no history of reaction for >2 years

• Detectable sIgE, no SPT available, does not eat nuts

• Discordance between SPT and sIgE, history of reaction

*Exclusion criteria for OFC*

• Parental consent refused

• Anaphylactic reaction after contact with peanut + peanut SPT ≥8 mm and/or peanut specific IgE ≥15 kUa/L

• Current poorly controlled asthma

• Unwell on the day of the challenge

*Recipe for peanut and placebo brownies.* Active and placebo brownies were baked on separate days, with separate utensils, to avoid cross-contamination. They were identical in appearance, smell, taste, viscosity, texture, structure, and volume. Sensory evaluation by individuals not participating in the study confirmed no differences between placebo and active brownies. The peanut flour (Golden Peanut Company, Alpharetta, Ga, USA) was constituted of partially defatted (12% fat) light-roasted peanut flour from runner-type peanuts (product code 521271).

*Procedures*. All peanut challenges were carried out at the Paediatric Day Care Unit at the University Hospital of South Manchester by the same personnel (N.N. and G.K.).

Participants with asthma were asymptomatic in the week before the challenge and had not used any bronchodilators in the last 24 hours before challenge. Subjects with seasonal allergic rhinitis were challenged outside the pollen season. Antihistamines were withheld for at least 72 hours before the challenge. On the challenge day, detailed medical history, physical examination, and baseline observations ensured that the subject was fit, and parental consent was obtained before proceeding with the challenge. Medication and equipment for resuscitation including oxygen and injectable adrenaline were readily available.

Challenges were not discontinued on the basis of subjective symptoms, but were stopped after development of 2 objective signs indicating an allergic reaction. Appropriate treatment was administered to those who had reacted. If no reaction had occurred, subjects were discharged after an observation period of 2 hours after the last given dose. Children who reacted were observed for at least 2 hours after their symptoms had resolved completely and were provided with a tailored management plan before discharge. In addition, they were contacted by phone in the evening and the following day by the study doctor to confirm that they were well and that no late-phase reactions had occurred.

*Open challenge*. Open challenges were performed in 4 stages and took approximately 4 hours. The first brownie contained 10 mg, the second 100 mg, the third 1 g, and the last 5 g peanut protein. The interval between stages was 30 minutes. Baseline observations (body temperature, oxygen saturation, pulse rate, blood pressure, and FEV_1_) were recorded before the administration of each dose and repeated every 30 minutes for as long as 2 hours after administration of the last dose.

Open challenges with roasted peanuts (1/8 of a peanut to 15 peanuts) followed similar intervals and observation protocol as those performed by using the peanut brownies.

*DBPCFC*. Double-blind placebo-controlled food challenges were performed in 10 stages (on the same day with 1 hour break between administration of placebo and active brownies) and lasted approximately 8 hours, including the 2-hour observation period after administration of the final dose. The active and placebo brownies were randomly allocated in 2 boxes labelled A and B; the code for unblinding was kept in a sealed envelope. Each box contained 5 brownies, and both the researchers and the child undergoing the challenge were unaware which box contained the active or placebo brownies. The first active brownie contained 1 mg peanut protein, the second 10 mg, the third 100 mg, the fourth 1 g, and the last 5 g peanut protein. The interval between stages and observations recording was the same as in the open challenge. The code was broken after the challenge was completed.

*Oral food challenge outcome*.

*Negative challenge:* no symptoms observed on OFC. Children with negative OFCs were considered peanut tolerant.

*Positive challenge:* Two or more objective signs, including flushing, pruritus, urticaria, angioedema, abdominal tenderness with increased bowel sounds, vomiting, diarrhoea, sneezing, rhinorrhoea, cough, hoarse voice, stridor, wheeze, >20% fall in FEV_1_, >30% drop in blood pressure, and loss of consciousness.

*Inconclusive challenge:* 1 objective sign or subjective symptoms only.

**Ara h 2 in characterisation of peanut allergic/peanut tolerant children**

Ara h 2 sIgE positivity was defined as Ara h 2 sIgE>0.35 kuA/L measured by ImmunoCAP and Ara h 2 sIgE>0.3 ISU measured by ISAC solid-phase allergen chip.

A total of four study participants who did not undergo OFC were diagnosed as peanut allergic based on Ara h 2 IgE positivity. Of those, two were sensitised at age 5 and/or 8 years, but had inconclusive double-blind placebo-controlled OFC (one sign/symptom). One of these children had no previous history of exposure, and another had a previous history of reaction documented at ages 3 and 8 years. A further 2 children were sensitised at ages 5 and 8 years, but their parents did not provide consent for a challenge. Both reported a history of reactions upon exposure, but had SPT MWD<8mm and/or peanut sIgE<15 kuA/L, and therefore did not qualify to be classified as peanut allergic without undergoing OFC.

Only one child with a (borderline) positive Ara h 2 level (0.38) at age 8 years had a negative challenge (DBPCC) and hence was classified as non-allergic. It is important to note that this child had an Ara h2 level <0.35 kuA/L at age 11 years.

**Definitions of variables**

**Parentally reported allergy to a specific food allergen:** Defined as a positive answer to the question referring to the specific food allergen: “Has your child had any problems/symptoms when eating peanuts, tree nuts, egg, milk, fish, sesame, or other food (specify)?

**Eczema at age 1 year:** Defined as a positive answer to the question “Did your doctor ever say that your child had eczema?” at the 1 year assessment

**Current eczema:** Defined as a positive answer to the question “Does your child have eczema now?”

**Ever eczema:** Defined as a positive answer to the question “Has your child ever suffered from eczema?”

**Age of eczema onset:** Participants were assigned into 4 categories according to the age of eczema onset: Never, 0-6months, 7-12months, and >12 months.

**Eczema Severity:** Defined according to parental report (interviewed), confirmed topical corticosteroid prescriptions from primary care records, and following clinical examination grading using the investigator global assessment (IGA) at the 3-year review, as:

1. **Mild:** dry skin, emollients only or emollients and mild steroids (creases only).
2. **Moderate:** more widespread and needing emollients and steroids.
3. **Severe:** infected/excoriated/bleeding, widespread, and needing steroid creams +/- antibiotics (topical or systemic).

**Current wheeze:** Defined as a positive answer to the question “Has your child had wheezing or whistling in the chest in the last 12 months?”

**Ever wheeze:** Defined as a positive answer to the question “Has your child ever had wheezing or whistling in the chest?”

**Doctor-diagnosed asthma:** Defined as a positive answer to the question “Has your doctor ever told you that your child has or had asthma?”

**Allergic sensitization:** Defined as a positive skin prick test (mean wheal diameter 3 mm larger than that elicited by the negative control to at least 1 of the allergens tested) or a positive sIgE (>0.35 kU_A_/L) to at least one allergen.

**Asthma:** Defined as the presence of any two of the following three features: current wheeze, current use of asthma medication, or physician-diagnosed asthma ever. Only those with none of these 3 features were classified as controls.

**Severe asthma and wheeze exacerbations:** Defined as a receipt of oral steroids for at least 3 days or emergency department visit or admission to hospital because of asthma and requiring oral steroid use.

**Wheeze Phenotypes** (29)

1. **No Wheeze:** never wheezed
2. **Transient Early Wheeze:** wheezed at any time <3y but no wheezing >3y
3. **Late-onset Wheeze:** No report of wheeze <3y but wheeze reported at age 5 or after
4. **Persistent Wheeze:** Wheeze at any time <3y and wheeze within the last 12 months reported at 5- & 8-year reviews.
5. **Intermittent Wheeze:** Wheezed at any one time point during the first 5 or at 8 years.

**Current rhinitis:** Defined as a positive answer to the question ‘‘Has your doctor ever told you that your child has hay fever or allergic rhinitis?’’ (age 1 and 3 y) or ‘‘In the past 12 months, has your child had a problem with sneezing or a runny or blocked nose when he/she did not have a cold or the flu?’’ (ages 5, 8, and 11 y).

**Data-driven phenotype allocation**

Cluster allocation of our study participants into latent atopy, wheeze, eczema, rhinitis, and atopic march/multimorbidity phenotypes, has previously been performed by MAAS study researchers using machine learning methods for latent variable analysis.

**Atopy Clusters** (30, 31)

1. **No atopic vulnerability:** Cluster characterized by few or no positive tests
2. **Predominantly non–dust mite:** Sensitivity mostly to allergens other than dust mite
3. **Predominantly House Dust Mite:** Sensitivity to mite but rarely to other allergens
4. **Multiple early:** Early sensitization to a broad panel of allergens
5. **Multiple late:** Late sensitization to a broad panel of allergens

**Atopic Multimorbidity Clusters** (32)

1. **No disease:** Children with low probability of eczema, wheeze, and rhinitis.
2. **Persistent Eczema & Late-Onset Rhinitis:** The prevalence of eczema increased steadily from 70% in early life to 95% at age 5y, with little resolution to age 11y. The probability of rhinitis increased to almost 100% by age 8y. These children had a low probability of wheeze throughout childhood.
3. **Eczema only:** High probability of eczema throughout life, peaking at 80% at age 5y, then declining steadily to a 50% probability at age 11y.
4. **Transient Wheeze:** High probability of wheeze within the first 5 y, with remission by age 8 y. Very low probability of eczema and rhinitis throughout childhood.
5. **Persistent Wheeze & Late-Onset Rhinitis:** High probability of wheeze throughout childhood, with increasing probability of rhinitis to almost 100% by age 11y. Probability of eczema was low, declining steadily to age 11y.
6. **Persistent Eczema & Wheeze:** Similar probability of wheeze and eczema throughout childhood, likely as co-morbidities, with a low probability of rhinitis throughout childhood.
7. **Rhinitis only:** Increasing probability of rhinitis from age 5 to 11y, but no wheeze or eczema.
8. **Atopic multimorbidity:** High probability of eczema from infancy to age 11y. The probability of wheeze increased with time. The probability of rhinitis increased from zero at age 1y to almost 100% by age 8y. Eczema developed first, followed by wheeze, and then rhinitis; there was little evidence of resolution of eczema by age 11y.

**Genotyping**

*FLG* genotyping was performed using probes and primers as previously described (33-35). Genotyping for R501X, S3247X and R2447X mutations were performed using a TaqMan based allelic discrimination assay (Applied Biosystems, Cheshire, UK). Mutation 2282del4 was genotyped by sizing of a fluorescent-labelled PCR fragment on a 3100 or 3730 DNA sequencer. *FLG* mutations 3673delC and 3702delG were assessed by GeneScan analysis of fluorescently labelled polymerase chain reaction products. Data were analysed as combined carriage of a *FLG* null allele, i.e., children carrying one or more of the six genetic variations were considered as having a *FLG* loss-of-function mutation (34). In cases with incomplete *FLG* data, the presence of one *FLG* mutation defined that case as a carrier; participants with incomplete genotyping data in whom all alleles successfully tested were wild type were excluded from further analysis, as it was not possible to determine their *FLG* genotype status (34).

**RESULTS**

5890 pregnant women attended 654 antenatal clinics. 1499 couples met inclusion criteria and gave consent for follow-up. 1184 babies were born into study and have at least some identifiable data. 1029 children attended follow up at 8y. 920 children had SPT for peanut and 605 children had sIgE for peanut. 936 children had either a SPT or sIgE for peanut and were considered for inclusion into study. 1 additional child, not tested at 8 years, was tested at age 5y, and 43 additional children were tested at age 11, so were also included into study.

A total of 13 (1.6%) and 26 (3.3%) children in study reported a previous reaction to peanuts at age 8y and 11y respectively. Only one child (0.1%) who had previously reported a reaction was classified as non-peanut allergic.

**Table S2:** Comparisons of demographic characteristics between included and excluded participants.

*denotes when p value is provided by Fisher’s exact test, Ref Var = reference variable

| Variables:  Categorical (proportions, %)  Numerical (mean, CI) | Whole Cohort  (n=1184) | Included  Participants (n=959) | Excluded  Participants  (n=225) | Incl. Vs Excl.  Chi-2  p-value |
| --- | --- | --- | --- | --- |
| Sex (Boys) | 642 (54.2%) | 514 (53.6%) | 128 (56.9%) | p=0.372 |
| Ethnicity (% Caucasian) | 1058 (95.0 %) | 895 (95.3%) | 163 (93.1%) | p=0.227 |
| Maternal age at birth  (in years) | 30.4 years  (30.1-30.7) | 30.6 years  (30.3-30.9) | 29.4 years  (28.8-30.1) | **p=0.002** |
| Gestational age at birth  (in weeks) | 39.9 weeks  (39.8-40.0) | 39.9 weeks  (39.8-40.0) | 39.7 weeks  (39.4-39.9) | p=0.076 |
| Socioeconomic class  Managerial  Intermediate  Routine  Not working | 464 (61.5%)  165 (21.9%)  94 (12.5%)  26 (3.5%) | 402 (61.6%)  151 (23.1%)  78 (11.9%)  17 (2.6%) | 62 (61.4%)  14 (13.9%)  16 (15.9%)  9 (8.9%) | Ref Var  p=0.099  p=0.351  **p=0.003** |
| Smoking in Pregnancy (Y)  Maternal  Paternal | 119 (11.6%)  287 (28.0%) | 109 (11.5%)  270 (28.6%) | 10 (12.7%)  17 (21.5%) | p=0.762  p=0.180 |
| Smoking, child’s age 1 year (Y)  Maternal  Paternal | 174 (14.8%)  312 (26.4%) | 129 (13.5%)  250 (26.1%) | 45 (20.2%)  62 (27.7%) | **p=0.012**  p=0.635 |
| Breast fed (Y) | 780 (70.0%) | 660 (71.7%) | 120 (61.9%) | **p=0.007** |
| Peanut Consumption (Y)  Before pregnancy  In pregnancy  During breastfeeding | 103 (92.8%)  91 (82.0)  69 (78.4%) | 88 (91.7%)  78 (81.2%)  59 (77.6%) | 15 (100%)  13 (86.7%)  10 (83.3%) | *p=0.595  *p=1.000  *p=1.000 |
| Pet ownership (Y)  Cat at recruitment (in pregnancy)  Cat at year 1  Dog at recruitment (in pregnancy)  Dog at year 1 | 240 (21.0%)  205 (20.0%)  201 (17.3%)  174 (16.9%) | 193 (20.6%)  190 (20.0%)  154 (16.4%)  163 (17.2%) | 47 (21.3%)  15 (19.2%)  47 (21.3%)  11 (13.9%) | p=0.814  p=0.867  p=0.085  p=0.459 |
| Older Sibling (Y) | 599 (55.7%) | 511 (53.6%) | 88 (71.5%) | **p=0.000** |
| Day care attendance (Y) | 741 (68.2%) | 629 (70.0%) | 112 (59.6%) | **p=0.005** |
| Parental atopy (Y)  Maternal  Paternal | 953 (82.9%)  682 (59.5%)  716 (62,8%) | 771 (82.7%)  542 (58.3%)  588 (63.8%) | 182 (83.9%)  140 (64.5%)  128 (59.5%) | p=0.686  p=0.096  p=0.246 |
| Parental asthma (Y)  Maternal  Paternal | 359 (30.3%)  235 (19.9%)  163 (13.8%) | 291 (30.3%)  187 (19.5%)  137 (14.3%) | 68 (30.2%)  48 (21.3%)  26 (11.6%) | p=0.971  p=0.535  p=0.293 |
| Eczema in the first year of life (Y)  Eczema severity at age 1 year  No eczema  Mild  Mod- Severe | 397 (36.4%)  414 (81.0%)  71 (13.9%)  26 (5.1%) | 318 (35.1%)  344 (82.7%)  54 (13.0%)  18 (4.3%) | 79 (42.5%)  70 (73.7%)  17 (17.9%)  8 (8.4%) | **p=0.058**  p= 0.097 |
| *FLG* Mutations (Caucasians only)  Any of the 6 mutations | 85 (10.6%) | 79 (10.7%) | 6 (9.2%) | p=0.711 |
| Allergic Sensitisation (by SPT)  +ve SPT to any allergen, age 1 year  +ve SPT to any allergen, age 3 years | 57 (11.3%)  225 (22.9%) | 46 (11.2%)  190 (22.8%) | 11 (11.7%)  35 (23.2%) | p=0.894  p=0.927 |
| Egg sensitisation (by SPT/sIgE)  Age 1 year  Age 3 years | 52 (10.4%)  53 (5.6%) | 43 (10.6%)  46 (5.7%) | 9 (9.7%)  7 (5.0%) | p=0.789  p=0.737 |
| Parentally reported wheeze  In first 1 year of life | 309 (28.0%) | 251 (27.4%) | 58 (30.7%) | p=0.364 |
| Parentally reported rhinitis  In first 3 year of life | 50 (4.6%) | 39 (4.3%) | 11 (5.9%) | p=0.344 |

**Table S3.** Multivariate logistic regression analysis of the predictors for peanut allergy: Analysis including maternal peanut consumption during breastfeeding among breastfeeding infants.

| Variables |  | | |
| --- | --- | --- | --- |
|  | aOR | 95% CI | p-value |
| Sex (Male) | 2.40 | 0.52-11.1 | 0.262 |
| Cat ownership in pregnancy | 2.94 | 0.63-13.7 | 0.170 |
| Eczema in the first year of life | 6.53 | 1.10-38.7 | 0.039 |
| Egg Sensitisation at age 3 years | 2.17 | 0.49-9.61 | 0.309 |
| Peanut consumption during breastfeeding | 1.16 | 0.22-6.04 | 0.864 |
| *FLG* loss-of function mutations | 1.83 | 0.38-8.76 | 0.450 |

**Figure S1:** Allergic sensitisation (SPT) in children with and without peanut allergy: a) Any allergen; b) Egg; c) Any inhalant allergen; d) Cat; e) House dust mite; f) Grass pollen

**Figure S2**: Eczema (a) and rhinitis (b) in children with and without peanut allergy

**Figure S3**: Current wheeze (a) and unscheduled GP (b) and A&E (c) visits with wheeze in children with and without peanut allergy

**Figure S4:** Doctor-diagnosed asthma (a) and use of asthma medication (b) in children with and without peanut allergy

**Table S4:** Asthma diagnosis at age 11 years in children with and without peanut allergy

| Variable  Categorical (proportions, %)  Numerical (mean, SD) | Study Population (n=959) | Not PA  (n=929) | PA  (n=30) | Chi-2  p value | Odds Ratio  (95% CI) |
| --- | --- | --- | --- | --- | --- |
| Asthma Doctor-Diagnosed  Age 11 years | 231 (27.3%) | 214 (26.2%) | 17 (58.6%) | p<0.001 | 3.99 (1.87, 8.48) |
| Asthma Diagnosis as per study criteria at age 11 years | 176 (23.8%) | 159 (22.3%) | 17 (65.4%) | p<0.001 | 6.58 (2.89, 15.0) |

**REFERENCES**

1. Tariq SM, Stevens M, Matthews S, Ridout S, Twiselton R, Hide DW. Cohort study of peanut and tree nut sensitisation by age of 4 years. Bmj. 1996;313(7056):514-7.

2. Ewan PW. Clinical study of peanut and nut allergy in 62 consecutive patients: new features and associations. Bmj. 1996;312(7038):1074-8.

3. Hourihane JO, Dean TP, Warner JO. Peanut allergy in relation to heredity, maternal diet, and other atopic diseases: results of a questionnaire survey, skin prick testing, and food challenges. Bmj. 1996;313(7056):518-21.

4. Eigenmann PA, Sicherer SH, Borkowski TA, Cohen BA, Sampson HA. Prevalence of IgE-mediated food allergy among children with atopic dermatitis. Pediatrics. 1998;101(3):E8.

5. Sicherer SH, Furlong TJ, Maes HH, Desnick RJ, Sampson HA, Gelb BD. Genetics of peanut allergy: a twin study. J Allergy Clin Immunol. 2000;106(1 Pt 1):53-6.

6. Lack G, Fox D, Northstone K, Golding J. Factors associated with the development of peanut allergy in childhood. N Engl J Med. 2003;348(11):977-85.

7. Hill DJ, Hosking CS, de Benedictis FM, Oranje AP, Diepgen TL, Bauchau V. Confirmation of the association between high levels of immunoglobulin E food sensitization and eczema in infancy: an international study. Clin Exp Allergy. 2008;38(1):161-8.

8. Du Toit G, Katz Y, Sasieni P, Mesher D, Maleki SJ, Fisher HR, et al. Early consumption of peanuts in infancy is associated with a low prevalence of peanut allergy. J Allergy Clin Immunol. 2008;122(5):984-91.

9. Fox AT, Sasieni P, du Toit G, Syed H, Lack G. Household peanut consumption as a risk factor for the development of peanut allergy. J Allergy Clin Immunol. 2009;123(2):417-23.

10. Sicherer SH, Wood RA, Stablein D, Burks AW, Liu AH, Jones SM, et al. Immunologic features of infants with milk or egg allergy enrolled in an observational study (Consortium of Food Allergy Research) of food allergy. J Allergy Clin Immunol. 2010;125(5):1077-83.e8.

11. Brown SJ, Asai Y, Cordell HJ, Campbell LE, Zhao Y, Liao H, et al. Loss-of-function variants in the filaggrin gene are a significant risk factor for peanut allergy. J Allergy Clin Immunol. 2011;127(3):661-7.

12. Tan HT, Ellis JA, Koplin JJ, Matheson MC, Gurrin LC, Lowe AJ, et al. Filaggrin loss-of-function mutations do not predict food allergy over and above the risk of food sensitization among infants. J Allergy Clin Immunol. 2012;130(5):1211-3.e3.

13. Du Toit G, Roberts G, Sayre PH, Plaut M, Bahnson HT, Mitchell H, et al. Identifying infants at high risk of peanut allergy: the Learning Early About Peanut Allergy (LEAP) screening study. J Allergy Clin Immunol. 2013;131(1):135-43.e1-12.

14. Koplin JJ, Allen KJ, Gurrin LC, Peters RL, Lowe AJ, Tang ML, et al. The impact of family history of allergy on risk of food allergy: a population-based study of infants. Int J Environ Res Public Health. 2013;10(11):5364-77.

15. Koplin JJ, Peters RL, Ponsonby AL, Gurrin LC, Hill D, Tang ML, et al. Increased risk of peanut allergy in infants of Asian-born parents compared to those of Australian-born parents. Allergy. 2014;69(12):1639-47.

16. Flohr C, Perkin M, Logan K, Marrs T, Radulovic S, Campbell LE, et al. Atopic dermatitis and disease severity are the main risk factors for food sensitization in exclusively breastfed infants. J Invest Dermatol. 2014;134(2):345-50.

17. Brough HA, Simpson A, Makinson K, Hankinson J, Brown S, Douiri A, et al. Peanut allergy: effect of environmental peanut exposure in children with filaggrin loss-of-function mutations. J Allergy Clin Immunol. 2014;134(4):867-75.e1.

18. Du Toit G, Roberts G, Sayre PH, Bahnson HT, Radulovic S, Santos AF, et al. Randomized trial of peanut consumption in infants at risk for peanut allergy. N Engl J Med. 2015;372(9):803-13.

19. Martin PE, Eckert JK, Koplin JJ, Lowe AJ, Gurrin LC, Dharmage SC, et al. Which infants with eczema are at risk of food allergy? Results from a population-based cohort. Clin Exp Allergy. 2015;45(1):255-64.

20. Panjari M, Koplin JJ, Dharmage SC, Peters RL, Gurrin LC, Sawyer SM, et al. Nut allergy prevalence and differences between Asian-born children and Australian-born children of Asian descent: a state-wide survey of children at primary school entry in Victoria, Australia. Clin Exp Allergy. 2016;46(4):602-9.

21. Grimshaw KE, Bryant T, Oliver EM, Martin J, Maskell J, Kemp T, et al. Incidence and risk factors for food hypersensitivity in UK infants: results from a birth cohort study. Clin Transl Allergy. 2015;6:1.

22. Perkin MR, Logan K, Tseng A, Raji B, Ayis S, Peacock J, et al. Randomized Trial of Introduction of Allergenic Foods in Breast-Fed Infants. N Engl J Med. 2016;374(18):1733-43.

23. Perkin MR, Logan K, Marrs T, Radulovic S, Craven J, Boyle RJ, et al. Association of frequent moisturizer use in early infancy with the development of food allergy. J Allergy Clin Immunol. 2021;147(3):967-76.e1.

24. Custovic A, Simpson BM, Murray CS, Lowe L, Woodcock A, Asthma NACM, et al. The National Asthma Campaign Manchester Asthma and Allergy Study. Pediatr Allergy Immunol. 2002;13(s15):32-7.

25. Ferris BG. Epidemiology Standardization Project (American Thoracic Society). Am Rev Respir Dis. 1978;118(6 Pt 2):1-120.

26. Pearce N, Weiland S, Keil U, Langridge P, Anderson HR, Strachan D, et al. Self-reported prevalence of asthma symptoms in children in Australia, England, Germany and New Zealand: an international comparison using the ISAAC protocol. Eur Respir J. 1993;6(10):1455-61.

27. Asher MI, Keil U, Anderson HR, Beasley R, Crane J, Martinez F, et al. International Study of Asthma and Allergies in Childhood (ISAAC): rationale and methods. Eur Respir J. 1995;8(3):483-91.

28. Nicolaou N, Poorafshar M, Murray C, Simpson A, Winell H, Kerry G, et al. Allergy or tolerance in children sensitized to peanut: prevalence and differentiation using component-resolved diagnostics. J Allergy Clin Immunol. 2010;125(1):191-7.e1-13.

29. Lowe LA, Simpson A, Woodcock A, Morris J, Murray CS, Custovic A, et al. Wheeze phenotypes and lung function in preschool children. Am J Respir Crit Care Med. 2005;171(3):231-7.

30. Simpson A, Tan VY, Winn J, Svensén M, Bishop CM, Heckerman DE, et al. Beyond atopy: multiple patterns of sensitization in relation to asthma in a birth cohort study. Am J Respir Crit Care Med. 2010;181(11):1200-6.

31. Lazic N, Roberts G, Custovic A, Belgrave D, Bishop CM, Winn J, et al. Multiple atopy phenotypes and their associations with asthma: similar findings from two birth cohorts. Allergy. 2013;68(6):764-70.

32. Belgrave DC, Granell R, Simpson A, Guiver J, Bishop C, Buchan I, et al. Developmental profiles of eczema, wheeze, and rhinitis: two population-based birth cohort studies. PLoS medicine. 2014;11(10):e1001748.

33. Bisgaard H, Simpson A, Palmer CN, Bonnelykke K, McLean I, Mukhopadhyay S, et al. Gene-environment interaction in the onset of eczema in infancy: filaggrin loss-of-function mutations enhanced by neonatal cat exposure. PLoS Med. 2008;5(6):e131.

34. Brough HA, Simpson A, Makinson K, Hankinson J, Brown S, Douiri A, et al. Peanut allergy: effect of environmental peanut exposure in children with filaggrin loss-of-function mutations. J Allergy Clin Immunol. 2014;134(4):867-75 e1.

35. Palmer CN, Irvine AD, Terron-Kwiatkowski A, Zhao Y, Liao H, Lee SP, et al. Common loss-of-function variants of the epidermal barrier protein filaggrin are a major predisposing factor for atopic dermatitis. Nat Genet. 2006;38(4):441-6.
